# High-efficiency electrochemical removal of tetracycline using a stainless steel electrode coated with activated chestnut shell biochar

**DOI:** 10.1039/d5ra05567a

**Published:** 2025-11-17

**Authors:** Jianan Zhang, Mengshuang Chai, Jinwei Zhang, Shijie Zhang, Shengrui Wang, Qin Zhou

**Affiliations:** a College of Advanced Agriculture and Ecological Environment, Heilongjiang University Harbin 150080 China zhouqin@hlju.edu.cn; b School of Chemistry and Materials Science, Heilongjiang University Harbin 150080 China

## Abstract

With the increasing discharge and improper disposal of antibiotics in the treatment of human diseases and in aquaculture, the widespread development of bacterial resistance has caused a serious public health problem. In this work, an activated chestnut shell biochar material (ACS) was coated onto stainless steel as the cathode and an iridium-tantalum titanium electrode used as the anode to investigate the electrocatalytic degradation of tetracycline in simulated wastewater. The results indicated that ACS pyrolyzed at 800 °C with 1 : 3 KOH has large specific surface area, uniform pore size, abundant active sites, and achieved the best catalytic performance to generate H_2_O_2_*via* oxygen reduction, where ˙OH plays an important role in the electrocatalytic degradation. Moreover, the removal efficiency of TC reached 90.6% in 200 min under the optimized conditions, as follows: an initial pH of 3, an applied current of 40 mA cm^−2^, and an initial concentration of 25 µg mL^−1^. In addition, it was found that the TC removal remained at about 86.77% even after 20 repeated degradation cycles. The electrocatalytic experiment verified that the cathode composed of the coated carbon material could accumulate about 1.8-times that of a bare-steel electrode through the 2e-oxygen reduction reaction (2e-ORR) in the same period. As a consequence, the ACS electrode showed high efficiency for the electrochemical degradation of tetracycline. The prepared materials have broad application prospects in the treatment of TC wastewater.

## Abstract 2

OH plays an important role in electrocatalytic degradation. TC removal remained at about 86.77% even after 20 repeated degradation cycles. The bare-steel electrode through the 2e-oxygen reduction reaction (2e-ORR).

## Graphical abstract

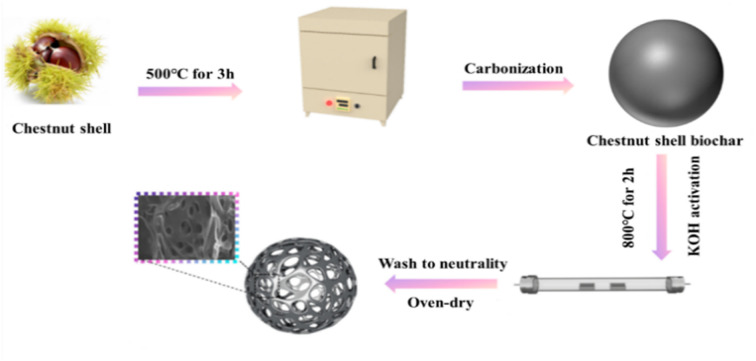

## 1. Introduction

The supply of adequate and safe water is an important factor in ensuring the well-being of humans. However, nowadays, with the abuse of antibiotics in the treatment of human diseases and aquaculture, the increased antibiotic contamination in water poses a threat to human health and safety of the ecosystem.^1,2^ Antibiotic residues are often detected at trace levels in various environmental substrates such as urban sewage, surface water, and groundwater.^3^ Tetracycline (TC) is one of the most widely used antibiotics in the world. A survey showed that antibiotic residues in water mainly come from industrial wastewater, aquaculture wastewater, medical waste, *etc.* containing TC.^4^ Traditional wastewater treatment plants cannot eliminate the presence of antibiotics. TC is readily soluble in water, possesses high chemical stability and a low rate of biodegradation, and is difficult to decompose naturally by traditional organisms.^5^ Therefore, the degradation of tetracycline has attracted extensive attention locally and abroad.

The traditional methods for the removal of tetracycline in wastewater mainly include photodegradation, chemical oxidation, bioremediation, electrochemical and adsorption methods. However, the large-scale application of these methods is often limited by their high cost and low efficiency. For instance, adsorption, though popular for its high removal efficiency, merely relocates the tetracycline residue within adsorbents and requires further processing of the loaded adsorbents, which are classified as hazardous waste.^6^ The electrochemical oxidation process is widely used because of its low cost and effectiveness. Compared with other systems, it has distinct advantages in terms of material cost and energy consumption. For example, compared with the PMS oxidation system, the electrochemical oxidation process does not require the addition of expensive PMS reagents. In contrast to the Fenton system, it avoids the production of secondary pollutants such as iron sludge.^7^ Compared with the ozone oxidation system, it does not need high energy-consuming ozone generation equipment. EAOPs, one type of electrochemical oxidation process, are an effective method to degrade TC.^8^ The principle of EAOPs is that pollutants are directly decomposed after contact with an electrode or their structure is destroyed after contact with the strong oxidant generated by the electrode, finally achieving the purpose of decomposition. In the oxidation process at the anode,^9,10^ organic pollutants are directly oxidized through electron exchange on the surface of the anode material without involving other substances, or through the oxidation species produced on the anode surface. At the cathode, the two-electron reduction of oxygen to H_2_O occurs,^11^ which in turn produces hydroxyl radicals to degrade organic matter. EAOPs have the advantages of environmental protection, low cost, and high pollutant degradation efficiency.^12^ Thus, the selection of electrode materials plays the most important role in the performance and degradation effect of EAOPs.^13^

As a renewable resource, biomass can generate thermochemical energy and high-performance materials at the same time. Biomass not only can be used to prepare porous carbon materials that have a large specific surface area but also effectively reduce their production cost and improve their electrical conductivity, and consequently the production of high-quality electrode materials.^14,15^ Therefore, researchers locally and abroad have conducted in-depth scientific research on porous carbon materials derived from biomass. Zhou *et al.*^16^ used tea as a biomass carbon source and synthesized the three-dimensional structure of tea porous carbon (TPC) *via* the template-free method. Yuan *et al.*^3^ used a bimetallic modified peanut shell-derived biochar catalyst to activate PS to degrade TC, and the removal efficiency reached 90% within 120 min. Zhou *et al.*^17^ investigated the adsorption performance of tetracycline using magnetic biochar modified by alkali and acid treatments. Their experimental results demonstrated that the modified biochar exhibited an enhanced adsorption efficiency. Moreover, the distribution of adsorption site energies was found to significantly influence its overall adsorption performance. Du *et al.*^18^ conducted a comprehensive review that thoroughly examined the advancements in the adsorption of antibiotics and antibiotic resistance genes (ARGs) from water using biochar and its modified materials. The article summarized the impacts of various modification methods on the adsorption performance of biochar and discussed its potential applications in environmental remediation. Xin *et al.*^19^ used a nitrogen/oxygen biomass self-doped porous carbon (NO/PC) cathode catalyst coupled with CuFeO_2_/biochar particle catalyst to degrade TC. Kim *et al.*^20^ employed asphalt as a carbon source and mesoporous SiO_2_ as a template and removed the template through KOH activation. At the same time, chemical etching resulted in the formation of activated carbon with multistage pore structures including large, medium, and micropores. Zheng *et al.*^21^ used plantain, a herb with nanometre, micron, and millimetre structures, as a carbon precursor, and prepared carbon materials with multilevel structures through carbonization activation. Zelic *et al.*^22^ reported the fabrication of hierarchical porous carbon nanosheets with an ultrahigh specific surface area of 3500 m^2^ g^−1^*via* a molten-salt templating strategy (NaCl/KCl), followed by KOH activation. The resulting material exhibited a narrow mesopore distribution centred at 3–10 nm and delivered an energy density of 65 Wh kg^−1^ at 4.5 V in an ionic-liquid electrolyte. Fajardo-Puerto *et al.*^23^ conducted an in-depth investigation into the enhancement of the Fenton process for antibiotic degradation using biochar–manganese composites, with a particular focus on the three-electron oxygen reduction reaction (ORR) pathway and its catalytic mechanisms. Their findings provide valuable insights into the development of advanced oxidation processes for environmental remediation. Li *et al.*^24^ conducted a comprehensive investigation into the kinetics and underlying mechanisms of the electrochemical transformation of chlorinated aromatic compounds using Fe-ZSM-5 as a catalyst. Their study revealed that Fe-ZSM-5 significantly enhanced both the reduction and oxidation reactions. The active sites within the Fe-ZSM-5 catalyst facilitated electron transfer, thereby accelerating the reaction rates. This finding not only elucidates the crucial role of Fe-ZSM-5 in environmental remediation and chemical synthesis but also provides a robust foundation for future research on the electrocatalytic degradation of pollutants.

Based on prior research, it has been demonstrated that the electrocatalytic performance of activated carbon materials can be significantly enhanced through KOH doping. In this context, chestnut shells have emerged as a low-cost and abundantly available material, exhibiting remarkable electrocatalytic activity.^25^ Chestnut shells, which are the residual byproducts after the extraction of chestnut kernels, are widely prevalent in China as a form of natural agricultural solid waste. These shells are naturally rich in nitrogen, a property that can markedly enhance the electrocatalytic performance of biochar. Specifically, the nitrogen content within chestnut shells can catalyse the formation of active sites on the surface of biochar, thereby improving its catalytic efficiency.^26^ The inherent structure of chestnut shells facilitates the production of biochar characterized by a high surface area and porosity. These attributes are crucial for enhancing the adsorption capacity and accessibility of the active sites, which are fundamental in achieving efficient electrocatalysis. Additionally, the intrinsic properties of chestnut shells contribute to the production of biochar with commendable thermal and chemical stability.^27^ This stability is essential for maintaining a consistent electrocatalytic performance over extended periods. In contrast, although rice husks are also abundant and low-cost, they typically exhibit a lower nitrogen content compared to chestnut shells. This disparity can impede the formation of nitrogen-doped active sites, which are advantageous for electrocatalysis. Moreover, the surface area and porosity of rice husk biochar may not be as pronounced as that of chestnut shell biochar, thereby affecting its adsorption and catalytic capabilities.^28^ Sawdust, another frequently utilized biomass material for the production of biochar, generally lacks the nitrogen content found in chestnut shells. The surface chemistry of sawdust biochar may also differ, resulting in less effective interactions with pollutants.^29^ Furthermore, the porosity and surface area of sawdust biochar may not be as optimized for electrocatalytic applications as that of chestnut shell biochar. Compared to other common biomass precursors such as rice husk and sawdust,^25,26^ chestnut shells are naturally rich in nitrogen and specific organic components, which facilitate the creation of biochar with an ultra-high specific surface area, abundant porosity, and abundant surface functional groups upon pyrolysis and activation, making it particularly promising for electrocatalytic applications.

At present, studies on the electrocatalytic degradation of tetracycline (TC) using chestnut shells are limited. In this study, biochar catalysts were prepared from chestnut shells at different pyrolysis temperatures to remove TC from wastewater, with the aim of identifying the optimal biochar for TC degradation and providing a basis for the treatment of antibiotics and an effective utilization pathway for chestnut shell waste. Chestnut shells contain a large number of proteins, sugars, and other components, which can promote the redox reactions of carbon electrodes, resulting in enhanced oxygen reduction properties, making chestnut shells a promising carbon source. In this study, chestnut shells were used as the carbon precursor for the cathode material to promote the TC degradation efficiency. Chestnut shell biochar (CS) was first prepared by preliminary carbonization in a muffle furnace, followed by KOH activation to obtain activated chestnut shell biochar (ACS). The electrocatalytic degradation system for TC was constructed using ACS-coated stainless steel mesh as the cathode and a commercially available iridium-tantalum-titanium (Ir–Ta–Ti) electrode as the anode. The process and effect of the electrochemical reaction on ACS were regulated by altering the current density, electrolyte concentration, and pH value. The most suitable cathode was selected for further investigation after being characterized and compared using scanning electron microscopy (SEM), Raman spectroscopy, and X-ray photoelectron spectroscopy (XPS). The removal mechanism of pollutants was thoroughly explored, and it was confirmed that the ACS material exhibited excellent electrocatalytic properties.

## 2. Experimental section

### 2.1 Materials and instruments

#### 2.1.1 Materials

The following reagents were utilized in this study, each procured from reputable manufacturers and adhering to stringent quality standards. PTFE emulsion with a concentration of 60% was obtained from Bide Chemical Reagent Co., Ltd. Anhydrous ethanol (A.R. grade) was provided by Tianjin Kemio Chemical Reagent Co., Ltd. Tetracycline (A.R. grade) was procured from Aladdin Reagent (Shanghai) Co., Ltd., while acetylene black (A.R. grade) was obtained from Li Zhiyuan Battery Sales Department. Stainless steel mesh with a mesh size of 40 was supplied by Changzhou Anhe Metal Products Co., Ltd. Phthalic acid (A.R. grade) was sourced from Macklin, and potassium hydroxide (A.R. grade) was obtained from Luoen Reagent. Sodium hydroxide (A.R. grade) and hydrochloric acid (A.R. grade) were both procured from Tianjin Guangfu Technology Development Co., Ltd. Polyethylene glycol (A.R. grade) and sodium sulfate (A.R. grade) were obtained from Macklin. Deionized water (A.R. grade) was prepared in-house. Potassium permanganate (A.R. grade) was sourced from Tianjin Kemio Chemical Reagent Co., Ltd. These reagents were carefully selected to ensure the precision and reliability of the experimental procedures.

#### 2.1.2 Instruments

The following instruments were used in this study: an electronic balance (JA2003N, Shanghai Precision Scientific Instrument Co., Ltd.), a heating magnetic stirrer with constant temperature (DF-101S, Zhengzhou Great Wall Scientific and Industrial Trade Co., Ltd.), a centrifuge (TGL-16 G, Hunan Xingke Instrument Co., Ltd.), a tubular resistance heating furnace (SK-3-10Q, Harbin Chengye Heat Treatment Equipment Manufacturing Co., Ltd.), a constant temperature magnetic stirrer (HJ-3, Changzhou Guohua Electric Co., Ltd.), an electrochemical workstation (CHI700e, Shanghai Chenhua Instrument Co., Ltd.), a fluorescence spectrophotometer (RF5301PC, Shimadzu Corporation, Japan), and an ultraviolet spectrophotometer (TUV-2250, Beijing Puxi General Instrument Co., Ltd.). These instruments were essential for conducting the experiments and obtaining precise measurements.

### 2.2 Material preparation

#### 2.2.1 Synthesis of ACS

**Fig. 1:**
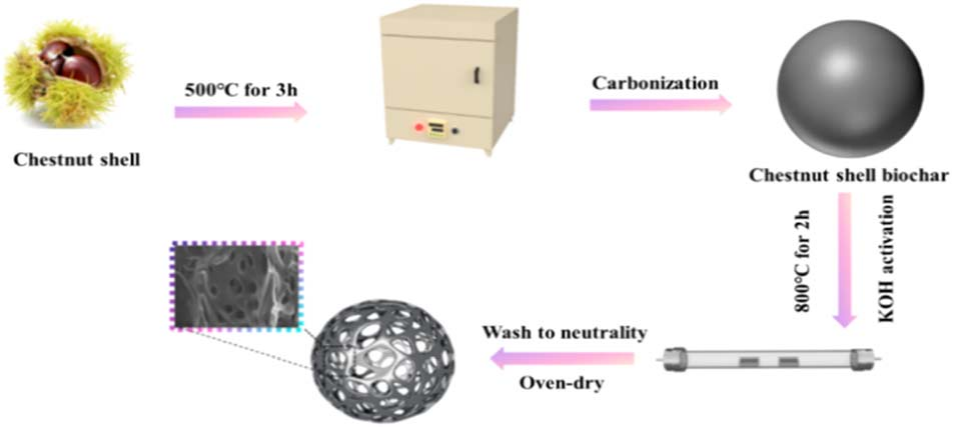
The synthetic route for ACS materials.

The activated carbon materials (ACS) were synthesized *via* chemical activation using chestnut shell biowaste as sustainable precursors. The chestnut shells were first pretreated by soaking in deionized water for 60 min to effectively remove impurities, and then washed and dried for subsequent use. Afterwards, they were mechanically stirred and calcined in a muffle furnace at 500 °C for 3 h to prepare the CS material. To get ACS, different ratios of KOH (1 : 1, 1 : 2, 1 : 3, 1 : 4; and 1 : 5) and CS were ground and mixed. The mixture was carbonized in a nitrogen-protected atmosphere at a heating rate of 5 °C min^−1^ at 700 °C, 800 °C, and 900 °C, respectively, for 2 h, and then naturally cooled to room temperature in a protective atmosphere. Finally, the activated porous biochar samples were washed several times with deionized water and ethyl alcohol to remove the residuals, and then dried at 80 °C to remove the moisture. The obtained activated biochar was labelled as ACS-*X-Y*, where ‘*X*’ denoted the carbonization temperature and ‘*Y*’ indicated the ratio of KOH to CS. The synthetic route for ACS is briefly described in Fig. 1.

#### 2.2.2 Preparation of ACS cathode materials

In this work, the electrocatalytic degradation system consisted of 304 stainless steel (3 cm × 3 cm) as the supporting current collector. The stainless steel was first carefully pretreated by soaking and sonicating it in acetone for 30 min. It was then soaked and sonicated in solutions containing 1 mol L^−1^ HCl and 1 mol L^−1^ Na_2_CO_3_ for 30 min each. To guarantee neutrality, it was lastly thoroughly cleaned several times with ethanol and water.^30^ Following treatment, the stainless steel was vacuum-dried in preparation for use. To make it usable, commercial PTFE emulsion with a 60% concentration was diluted to 10%. A mixture was created by combining 0.5 g of ACS, 1.75 g of polytetrafluoroethylene, and an adequate amount of anhydrous ethanol in a beaker. In a water bath set at 60 °C, this mixture was swirled until the water evaporated and a paste was formed. After that, the paste was repeatedly pressed onto a 3 cm × 3 cm piece of stainless steel mesh to guarantee a sturdy shape. For greater stability, the prepared electrode was cooked in deionized water for 30 min before being dried before use.

### 2.3 Electrochemical degradation of tetracycline using ACS cathode material

In the industrial wastewater simulation, the tetracycline concentration was maintained at 25 mg L^−1^. The generated ACS electrode served as the cathode in the electrochemical degradation system, while an iridium–tantalum–titanium mesh served as the anode. The electrolyte solution was sodium sulphate at 0.1 M. An adjustable DC power supply (MT1520) provided a constant current for the electrochemical breakdown of tetracycline. The current density was varied at 25, 30, 35, 40, and 45 mA cm^−2^, and the pH value was modified to 3, 5, and 7. The variations in tetracycline concentration were monitored using a UV spectrophotometer calibrated at 357 nm.^31^ All degradation experiments were performed in triplicate, and the average values along with standard deviations are reported.

### 2.4 Characterization

In this investigation, a scanning electron microscope (SEM, Crossbeam 540, Germany) was used to examine the morphologies and microstructures of the activated chestnut shell carbon materials. Peak fitting and an analysis of the surface elemental chemical states of the carbon materials were conducted using X-ray photoelectron spectroscopy (XPS, DXR3, USA). By examining the D band (defects or disordered phase) and G band (in-plane bond vibrations), Raman spectroscopy was utilized to identify the graphite and amorphous phases of the carbon materials. A laser confocal microscope Raman spectrometer (Thermo Scientific DXR3, USA) in the wavenumber range of 800–2000 cm^−1^ was used for the studies. The elemental makeup and surface functional groups of the ACS samples were thoroughly examined using XPS (AXIS Supra+, UK). The chemical composition and structural features of the carbon materials were identified by detecting their functional groups and chemical bonds using Fourier-transform infrared spectroscopy (FT-IR, 1725-X, USA). The pore size analysis was conducted using the Barrett–Joyner–Halenda (BJH) method, which has been proven to be highly effective in describing micropores, mesopores, and macropores because of its exceptional precision. This technique offers detailed information about the pore types and distribution properties of materials, in addition to enabling accurate pore size classification. The electrochemical characteristics of the materials were assessed using a CHI700e electrochemical workstation and cyclic voltammetry (CV), a useful technique for identifying redox peaks. To fully cover the impedance properties across various frequencies, electrochemical impedance spectroscopy (EIS) measurements were conducted over the frequency range of 100 kHz to 0.01 Hz. To provide thorough data support for studying the resistive properties of the materials, this frequency range was chosen to capture their electrochemical responses at different time scales.

## 3. Results and discussion

### 3.1 Material characterization analysis

**Fig. 2:**
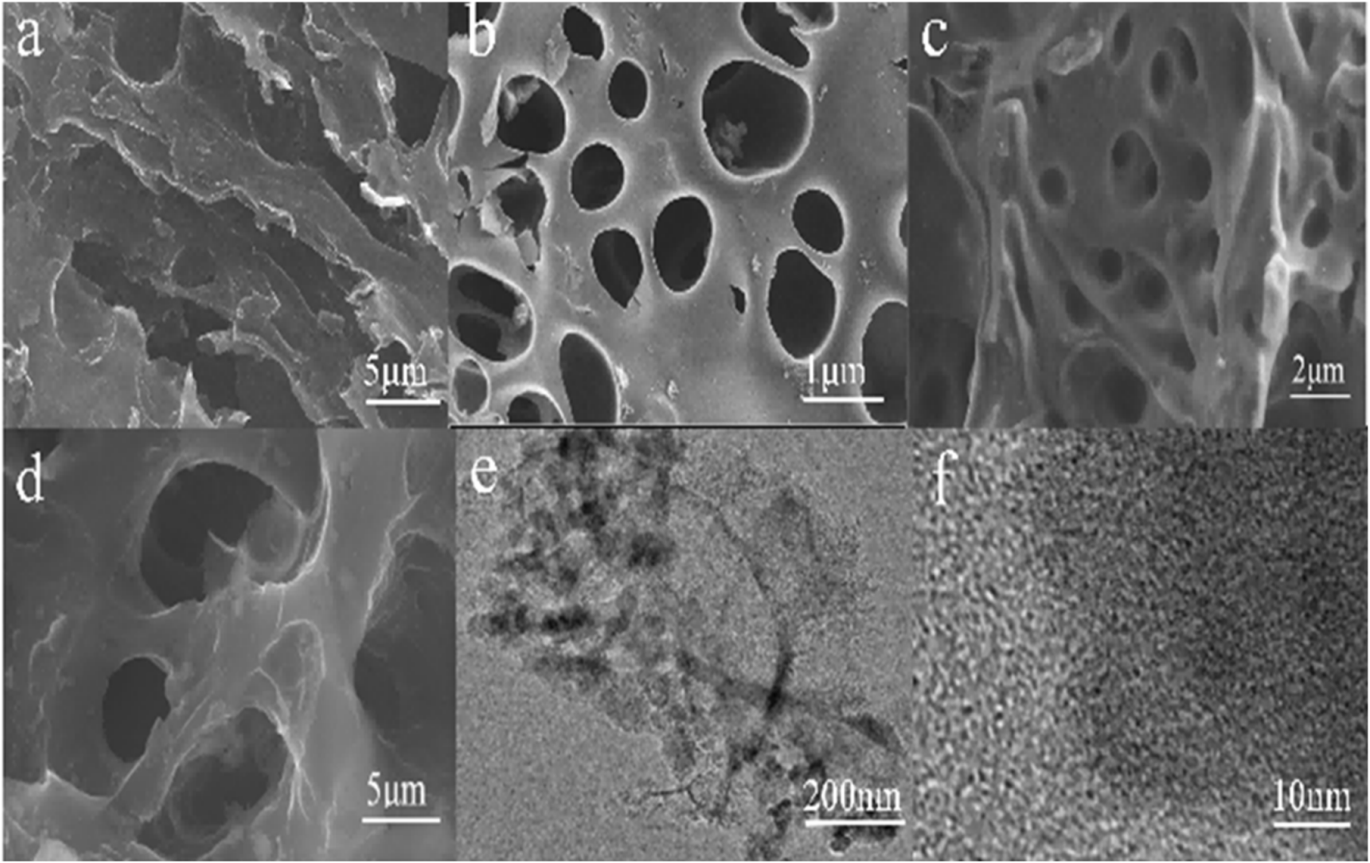
SEM images of CS (a) and ACS-800-3 (b–d), TEM image of ACS-800-3 (e), and HRTEM image of ACS-800-3 (f).

Analysis using scanning electron microscopy (SEM) showed that the surface morphological properties of the carbon materials varied significantly. Distinct surface grooves were seen in the pristine, unaltered chestnut shell (CS), as seen in Fig. 2(a), demonstrating the preservation of the skeletal characteristics of the original carbon material.^32^ Alternatively, following KOH activation treatment, the ACS-800-3 sample showed a large number of microporous and mesoporous structures (Fig. 2(b–d)), with the micropores substantially outnumbering the mesopores, which is in line with the findings of the pore size distribution.^33^ The fact that this porous structure was intact and widely dispersed suggests that the etching process successfully increased the specific surface area and internal structure of this material. Significant morphological alterations between the chestnut shell before and after treatment were discovered through additional research. The initial structure of the raw material was disturbed throughout the treatment process by the breakdown and separation of cellulose, lignin, and hemicellulose. Nevertheless, its specific surface area and internal porosity greatly increased by the new active sites created by the collapsed regions. The electrochemical performance of the material was eventually improved by this structural modification, which increased its reactive surface area and enhanced electron transfer efficiency.

**Fig. 3:**
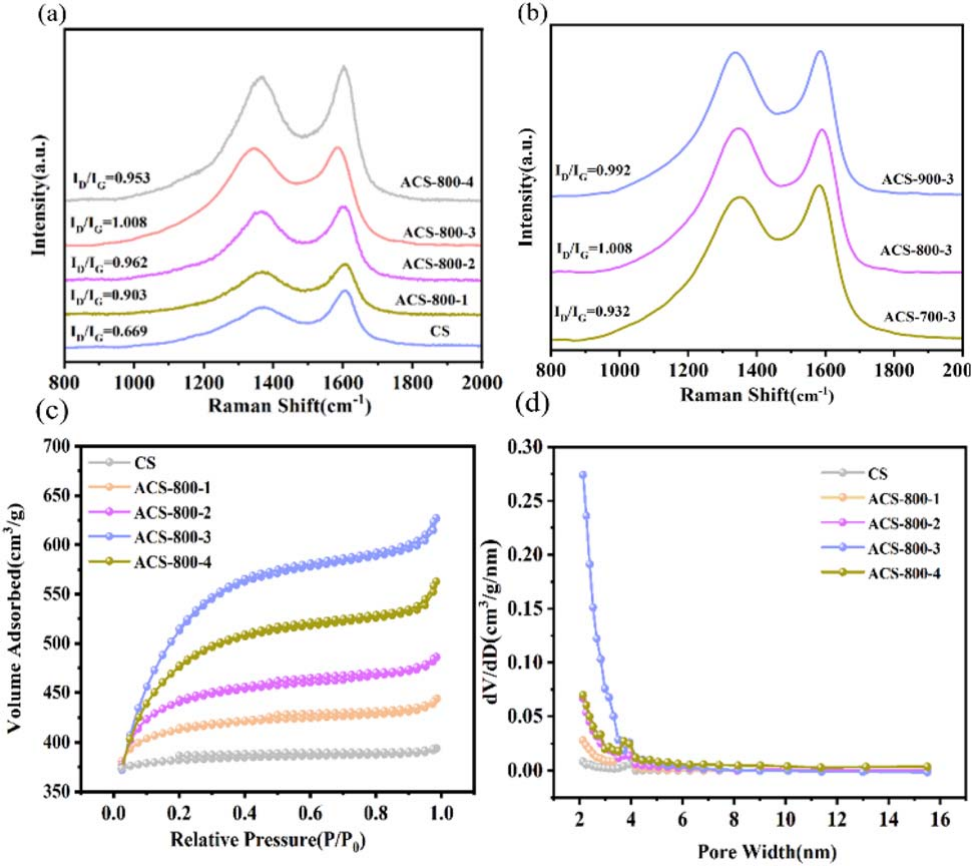
Raman scattering spectra of CS and ACS-800-*Y* (*Y* = 1–4) (a) and ACS-*X*-3 (*X* = 700–900) (b). N_2_ adsorption–desorption isotherms (c) and pore size distributions (d) of CS and ACS-800-*Y* (*Y* = 1–4).

Enhancing the electrochemical activity of carbonaceous materials is a well-established technique that involves defect engineering.^34^Fig. 3(a and b) display the Raman scattering spectra of the CS materials. A disorder-related peak at 1348 cm^−1^ (D band) and another peak at 1580 cm^−1^ (G band) are visible in the Raman scattering spectra.^35,36^ The G band at 1580 cm^−1^ indicates the existence of sp^2^-hybridized carbon in the graphite layers, while the D band at 1348 cm^−1^ is linked to structural flaws. One important metric for evaluating the degree of graphitization and defect in materials is the *I*_D_/*I*_G_ ratio.^37^ A higher *I*_D_/*I*_G_ value indicates a more faulty and less graphitized material. The ACS materials activated with varying KOH ratios exhibit higher *I*_D_/*I*_G_ values than CS, which has an *I*_D_/*I*_G_ value of 0.669. The highest *I*_D_/*I*_G_ value was achieved by ACS-800-3. The *I*_D_/*I*_G_ values of ACS-700-3, ACS-800-3, and ACS-900-3 are 0.932, 1.008, and 0.81 at different calcination temperatures, respectively. Among them, owing to its highest defectiveness, ACS-800-3 should have the highest catalytic activity. By speeding up electron transmission, its many defect sites provide a large number of reaction sites and improve the catalytic efficiency. The high *I*_D_/*I*_G_ ratio of 1.008 for ACS-800-3 indicates a significantly defective carbon structure. This level of disorder is generally higher than that reported for many other biomass-derived carbons (*e.g.*, from rice husk or straw, which typically exhibit *I*_D_/*I*_G_ ratios below 1.0),^28^ which is beneficial for providing more active sites and facilitating electron transfer, crucial for enhancing the electrocatalytic performance. Therefore, ACS-800-3 is a better choice as a cathode electrode material for electrocatalysis.

Specific surface area and pore size distribution, key factors influencing the electrocatalytic performance of prepared materials, are also fundamental indicators of the physical properties of electrode materials. To investigate the specific surface area and pore structure of ACS, we measured its N_2_ adsorption–desorption isotherms. As shown in Fig. 3(c), the isotherms were classified according to the IUPAC recommendations. The CS sample exhibited a type I isotherm, characteristic of microporous materials. In contrast, all the ACS samples displayed a combination of type I and type IV(a) isotherms, with a distinct H4-type hysteresis loop observed in the relative pressure (*P*/*P*_0_) range of 0.4 to 0.8. This indicates that KOH activation successfully constructed a hierarchical pore structure within the ACS materials, which integrates a significant volume of micropores (responsible for the high initial uptake) with a considerable proportion of narrow slit-like mesopores (evidenced by the hysteresis loop).^38^ As the amount of KOH increased, the specific surface area and pore volume of ACS-800-3 gradually increased compared to CS, with the BET area increasing from 444.174 m^2^ g^−1^ to 1844.129 m^2^ g^−1^ and pore volume increasing from 0.015 cm^3^ g^−1^ to 0.295 cm^3^ g^−1^. However, when the mass ratio of KOH to CS increased to 1 : 4, the specific surface area and pore volume first increased, and then decreased, reaching the maximum values for ACS-800-3. This indicates that an appropriate amount of activator enhances the specific surface area of biochar materials. The experimental results confirm the feasibility of optimizing the structure of the CS material by varying the KOH ratio, achieving the optimal specific surface area and maximum pore volume. As summarized in Table 1, the ACS-800-3 sample exhibits an ultra-high specific surface area (1844.129 m^2^ g^−1^) and a large pore volume (0.295 cm^3^ g^−1^). These values are notably superior to that typically reported for biochar derived from other biomass such as rice husk (500–1000 m^2^ g^−1^)^28^ or sawdust (800–1500 m^2^ g^−1^).^29^ This highlights the unique advantage of chestnut shells as a precursor, in combination with our activation strategy, for constructing a highly developed porous structure, which provides an ideal platform for mass transport and exposure of active sites. The larger specific surface area facilitates surface catalytic reactions and promotes the adsorption of organic pollutants, thereby enhancing the efficiency for the electrocatalytic degradation of tetracycline.

**Table 1:** Pore characteristics of the CS, ACS-800-1, ACS-800-2, ACS-800-3, and ACS-800-4 materials prepared under different activation conditions

Samples	*S*_bet_ (m^2^ g^−1^)	*S*_micro_ (m^2^ g^−1^)	*V*_pore_ (m^3^ g^−1^)	Pore width_aver_ (nm)
CS	444.174	17.634	0.015	2.120
ACS-800-1	1289.039	51.223	0.054	2.127
ACS-800-2	1682.946	93.067	0.092	2.124
ACS-800-3	1844.129	346.200	0.295	2.126
ACS-800-4	1826.846	222.497	0.104	2.130

**Fig. 4:**
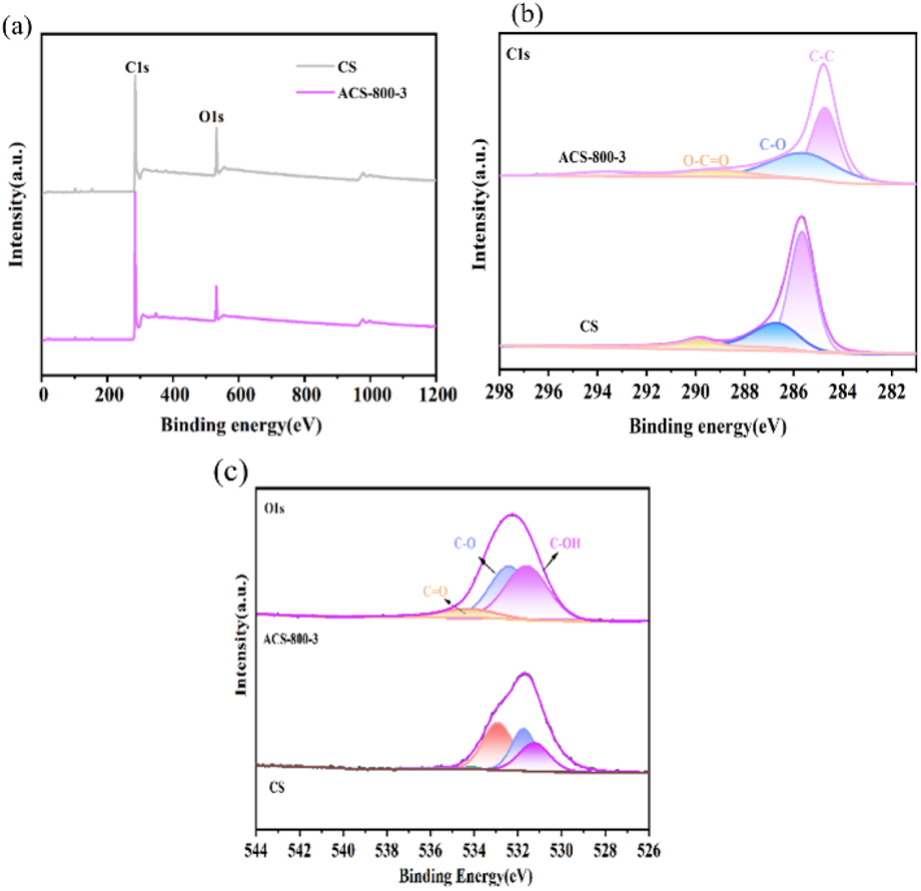
XPS spectrum of the ACS-800-3 material (a) and high-resolution XPS spectra of C 1s (b) and O 1s (c) for ACS-800-3.

X-ray photoelectron spectroscopy (XPS) provides information on the chemical bonds and oxidation states on the surface of a material, aiding in elucidating its catalytic mechanisms.^39^ As shown in Fig. 4(a), the spectra indicate that all the synthesized samples contain C and O. In the case of the chestnut shell-derived porous carbon, its C1s spectrum (Fig. 4(b)) shows peaks at binding energies of 284.6, 285, and 289 eV, corresponding to C–C, C–O, and O–C=O bonds, respectively. Its O 1s spectrum (Fig. 4(c)) exhibits peaks at 531, 532, and 534 eV, attributed to bulk C–OH, C–O, and C=O bonds, respectively.^40^ No nitrogen heteroatoms were detected, likely due to the lower nitrogen content in the chestnut shells and their potential volatilization during high-temperature pyrolysis. This suggests that the enhanced electrocatalytic performance is primarily attributed to the high specific surface area and porous structure of the material rather than nitrogen doping. Further treatment with the strong base KOH resulted in a low nitrogen content in ACS. This low nitrogen characteristic makes ACS a promising non-metal electrocatalyst for efficient tetracycline degradation.

**Fig. 5:**
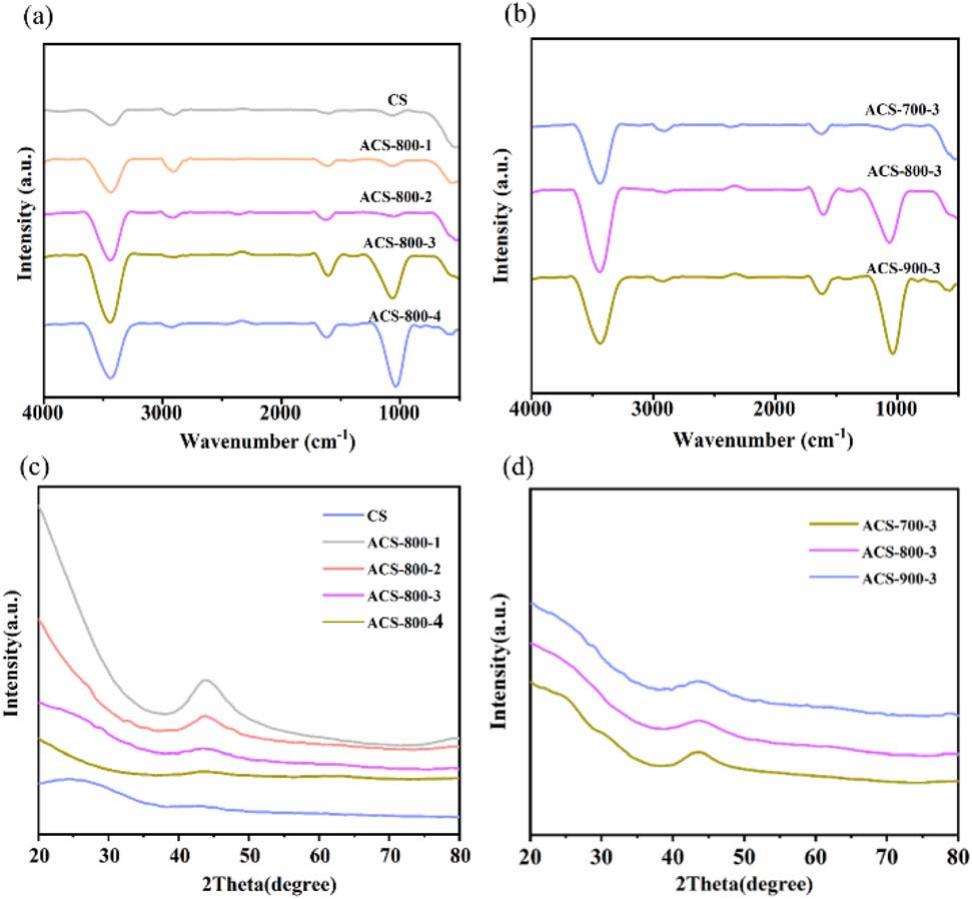
Fourier-transform infrared spectra of CS and ACS-800-*Y* (*Y* = 1–4) (a) and ACS-*X*-3 (*X* = 700–900) (b). XRD patterns of CS and ACS-800-*Y* (*Y* = 1–4) (c) and ACS-*X*-3 (*X* = 700–900) (d).

Fourier-transform infrared spectroscopy (FTIR) is employed to analyse the presence of functional groups and their chemical interactions within materials. As depicted in Fig. 5(a and b), the FTIR spectra illustrate the functional group characteristics and crystallographic structures of the carbon materials prepared using varying KOH activation ratios and different calcination temperatures, respectively. The absorption peak at 3438 cm^−1^ is typically attributed to the stretching vibrations of hydroxyl groups (R–OH). In the ACS-800-3 sample, this peak reaches its maximum intensity, indicating a higher content of hydroxyl groups. The elevated hydroxyl group content not only enhances the reactivity of the sample but also improves its hydrophilicity, as hydroxyl groups are quintessentially hydrophilic functional groups. Additionally, the absorption peak at 1619 cm^−1^ is associated with the stretching vibrations of aromatic C=C and C=O bonds.^41^ The characteristic absorption peak at 1113 cm^−1^ suggests the presence of abundant aromatic C–O groups on the surface and subsurface of the sample.^41^ These findings demonstrate that KOH activation significantly influences the surface chemistry of the carbon materials, providing a foundation for optimizing their performance in catalytic and adsorption applications. The abundant surface functional groups on ACS-800-3, as confirmed by XPS and FT-IR, not only enhance the hydrophilicity and electrolyte wettability of the electrode but are also likely to act as active sites, promoting electron transfer and the generation cycle of H_2_O_2_/˙OH. In contrast, biochar derived from many lignocellulosic or siliceous precursors often possess a lower diversity and density of these functional groups, further underscoring the superiority of chestnut shell-derived carbon in terms of surface chemistry for electrocatalysis.

The crystal structures of the carbon materials activated with varying KOH ratios and those exposed to different temperatures are depicted by their XRD patterns, as seen in Fig. 5(c and d), respectively. At 2*θ* = 23.5°, CS showed a diffraction peak attributed to the standard (002) plane of graphite. The samples showed a second peak at 43.7°, which corresponded to the (110) plane,^42^ after KOH pyrolytic activation, while the peak located at 23.5° vanished. This shows that the ordered structure of CS is broken by KOH activation, resulting in materials with less graphitization.^43^ The CS material structure is affected by the KOH ratio, and the addition of potassium broadens the peaks, changes the filling distances in the carbon materials, causes interlayer carbon disorder or flaws during activation, and releases a substantial amount of gas during KOH fusion. In contrast to CS, ACS exhibits decreased graphitization and increased electrochemical activity. The XRD data for the ACS materials treated at various calcination temperatures showed no discernible variations, suggesting that changes in the calcination temperature have no effect on the carbon form or graphite structure of this material.

### 3.2 Effect of reaction paraments on TC degradation

#### 3.2.1 Effect of KOH activation ratio

**Fig. 6:**
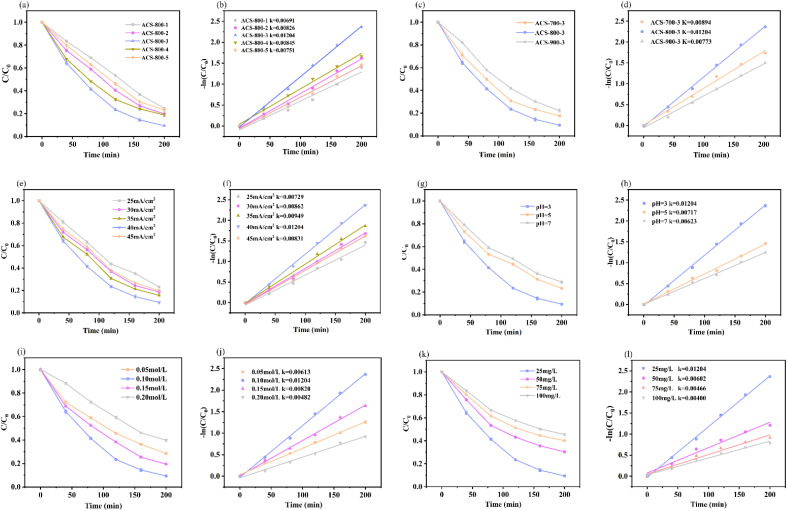
(a and b) Effect of ACS-800-*Y* (*Y* = 1–5) on degradation of tetracycline and corresponding kinetic curves; (c and d) effect of ACS-800-*Y* (*Y* = 1–5) on degradation of tetracycline and corresponding kinetic curves; (e and f) effect of current density on degradation of tetracycline and corresponding kinetic curves; (g and h) effect of pH value on the degradation of tetracycline and corresponding kinetic curves; (i and j) effect of electrolyte concentration on tetracycline degradation and corresponding kinetic curves; and (k and l) effect of tetracycline initial concentration on tetracycline degradation effect and corresponding kinetic curve.

The effects of different KOH mass ratios on electrocatalytic tetracycline (TC) breakdown were examined using pyrolysis tests carried out at 800 °C. As the KOH mass ratio increased from 1 : 1 to 1 : 3, the TC degradation efficiency dramatically increased from 60.8% to 90.6%, as seen in Fig. 6(a). Nevertheless, the degradation rate stabilized and decreased monotonically when the ratio was further increased to 1 : 5. According to the activation mechanism outlined in eqn (1-1) and (1-2), as follows:

1-1 KOH + C → K_2_CO_3_ + H_2_

1-2 K_2_CO_3_ → K_2_O + CO_2_

KOH etches the material surface to form porous structures during activation. Insufficient activator amounts lead to incomplete contact between the material and activator, resulting in partially inactivated biochar, which retains its original morphology, hindering electron transfer and electrocatalytic degradation.^43^ Excessive activation thins the pore walls, making them fragile and prone to destruction during subsequent processing, which compromises the pore stability. To optimize the activity and TC degradation efficiency of ACS, an appropriate KOH mass ratio is essential. ACS-800-3, prepared with a chestnut shell-to-KOH ratio of 1 : 3, achieved 90.6% degradation within 200 min, outperforming other materials. As shown in Fig. 6(b), this ratio also yielded the highest reaction rate constant (*k* = 0.01204 min^−1^), reinforcing the conclusion that a 1 : 3 ratio is optimal for activation.

#### 3.2.2 Effect of activation temperature

The activation effect is closely related to the calcination temperature. With a fixed ratio of chestnut shell (CS) to KOH, the impact of different activation conditions on tetracycline (TC) degradation was investigated. As shown in Fig. 6(c), the TC degradation rate increased significantly as the calcination temperature increased from 700 °C to 800 °C, but decreased when the temperature further increased to 900 °C. This confirms the significant influence of the calcination temperature on TC degradation. During activation, K_2_CO_3_ generated from the pyrolysis of KOH and carbonaceous materials undergoes two pyrolysis reactions. Changes in the gas release rate can affect the formation of pore structures, reducing the charge transfer efficiency among the cathode material, its surface, and the solution.^44^ The results showed that the CS cathode materials calcined at 800 °C exhibited the best electrocatalytic TC degradation performance. The kinetic analysis (Fig. 6(d)) further supports this, with the reaction rate constant peaking at 800 °C, indicating the optimal preparation temperature for high-performance ASC materials.

#### 3.2.3 Effect of current density

The electrocatalytic degradation of TC by ACS-800-3 was examined at different current densities, with the degradation curves presented in Fig. 6(e). The TC degradation efficiency increased from 65.7% to 90.6% as the current density increased from 25 mA cm^−2^ to 40 mA cm^−2^, but a further increase in the current density to 45 mA cm^−2^ led to a decline in the degradation efficiency. At low current densities, the utilization of catalytic active sites is limited, with only a portion of the sites participating in the reaction, resulting in a lower TC degradation efficiency. As the current density increases, the oxidation reaction rate at the electrode surface accelerates, promoting the generation of ˙OH radicals, which speeds up the degradation of organic pollutants. However, excessively high current densities increase the incidence of side reactions. These side reactions consume H_2_O and H^+^ within the electrolyzes, reducing the generation efficiency of ˙OH radicals, and thereby affecting the degradation of TC.^45^ The kinetic analysis (Fig. 6(f)) revealed that the reaction rate constant peaked at a current density of 40 mA cm^−2^. From the perspective of energy efficiency, selecting an appropriate current density is crucial for ensuring efficient and stable TC degradation. Therefore, a current density of 40 mA cm^−2^ was determined to be optimal for achieving efficient electrocatalytic TC degradation in this study.

#### 3.2.4 Effect of pH

The impact of different pH conditions on ACS-800-3-mediated TC degradation was investigated. TC solutions at pH 3, 5, and 7 were prepared in 0.1 mol L^−1^ Na_2_SO_4_. As shown in Fig. 6(g), the degradation efficiency decreased with an increase in pH. The pH affects ˙OH generation and indirectly determines the reaction efficiency. TC degradation relies on interactions with ˙OH radicals. At lower pH, a higher H^+^ concentration promotes ˙OH formation, enhancing the degradation of TC. Acidic conditions favour E-ORR (eqn (2-1)) and Fenton reactions,^46,47^ making them suitable for TC degradation.^48^ The kinetic analysis (Fig. 6(h)) showed the maximum reaction rate constant at pH 3, and thus selected as the optimal condition.

2-1 O_2_ + 2H^+^ + 2e^−^ → H_2_O_2_

#### 3.2.5 Effect of electrolyte concentration

The degradation performance of ACS-800-3 towards TC under different electrolyte concentrations was studied using sodium sulphate (Na_2_SO_4_), an inert electrolyte. As shown in Fig. 6(i), the electrolyte concentration significantly impacts the degradation efficiency. The degradation efficiency reached 90.6% at 0.1 mol L^−1^ Na_2_SO_4_, indicating an excellent catalytic performance. Both lower and higher electrolyte concentrations reduced the degradation efficiency. At low electrolyte concentrations, a reduced ionic content lowers the solution conductivity and current transfer efficiency. Given that current is a key driver of electrocatalytic degradation, a low transfer efficiency weakens the degradation driving force, reducing the efficiency. Moreover, a low electrolyte concentration may inadequately cover the catalyst surface, underutilize active sites and further reduce the efficiency. Conversely, although high electrolyte concentrations increase the conductivity, they also have negative effects. A high ionic strength can interfere with the catalyst-TC molecule interactions, making the degradation of TC difficult.^49^ Additionally, excessive electrolyte concentrations can form a “salt film” on the catalyst surface, blocking its active sites and the reducing degradation efficiency. The kinetic analysis (Fig. 6(j)) supports these findings, showing the maximum reaction rate constant at 0.1 mol L^−1^ Na_2_SO_4_. This concentration optimally enhances the conductivity without hindering the TC-catalyst contact, making it the optimal choice for efficient TC degradation.

#### 3.2.6 Effect of initial tetracycline concentration

The degradation performance of the ACS-800-3 electrodes was investigated across different initial TC concentrations. The experimental results (Fig. 6(k)) showed that the degradation rate decreased as the TC concentration increased from 25 mg L^−1^ to 100 mg L^−1^. The degradation efficiency declined with higher initial TC concentrations, while the concentration of degradation intermediates increased significantly. The efficiency of ˙OH generation by the ACS-800-3 electrodes remained constant. Consequently, as the TC concentration increased within the fixed reaction time, the competition between the intermediates and TC for ˙OH intensified, reducing the TC degradation rate. The kinetic analysis (Fig. 6(l)) revealed that the reaction rate constant was maximized at an initial TC concentration of 25 mg L^−1^. Based on this analysis, 25 mg L^−1^ was determined to be the optimal initial concentration for TC degradation.

### 3.3 Electrochemical performance of chestnut shell cathode material

**Fig. 7:**
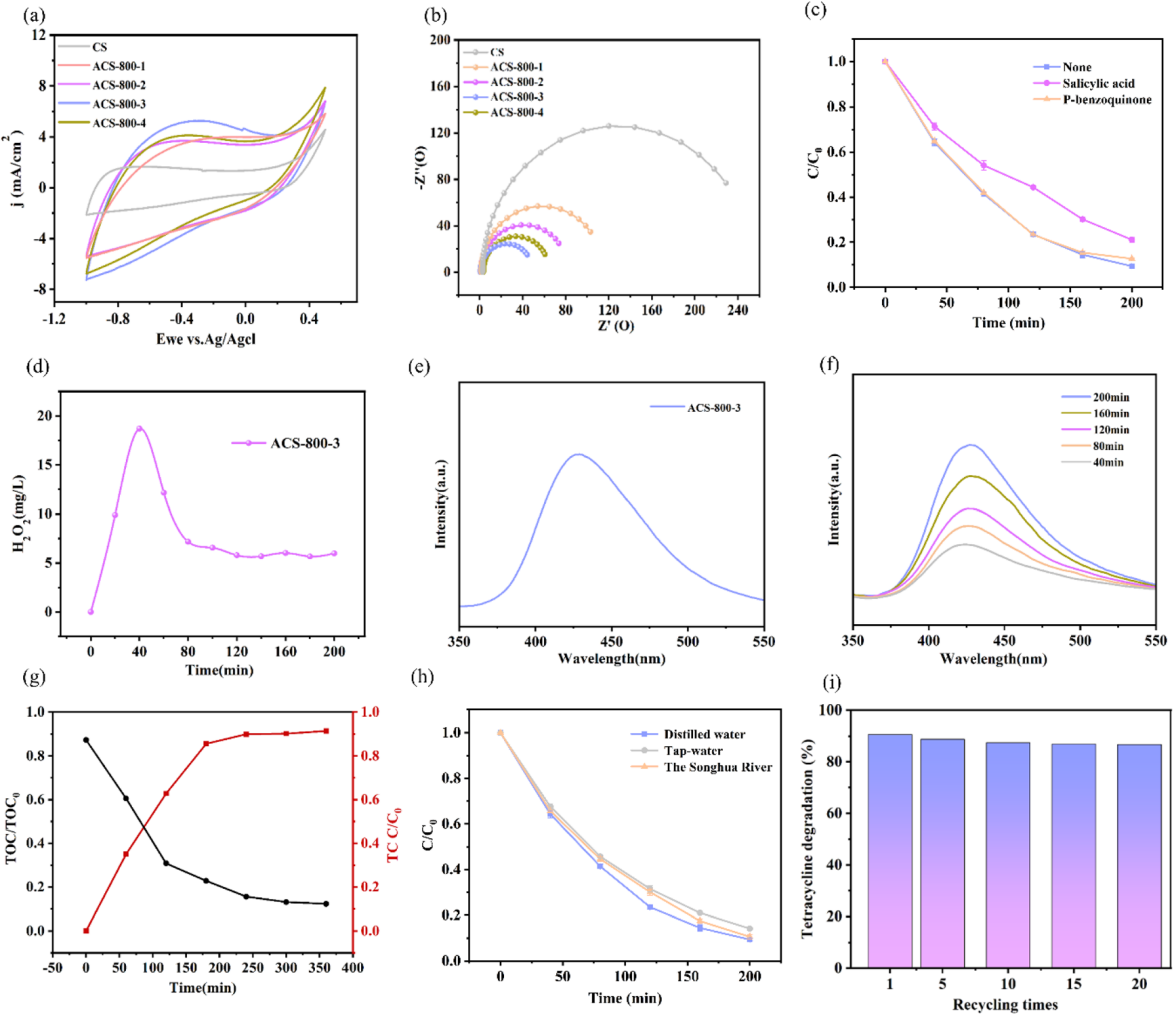
CV curve of the ACS-800-*X* (*X* = 0–4) electrode in 6 M KOH solution (a); EIS Nyquist curve of the ACS-800-*X* (*X* = 0–4) electrode in 6 M KOH solution (b); radical quenching experiments (c); H_2_O_2_ variation (d), fluorescence wavelength of ACS-800-3 for ˙OH production (e); fluorescence spectrum of ACS-800-3 for ˙OH production (f); TOC curves for ˙OH production over time (black: mineralization rate and red: degradation rate) (g), degradation efficiency of TC in different water matrices (h); and cyclic experiments (i).

To investigate and evaluate the impact of KOH doping on the electrocatalytic performance of the CS materials, cyclic voltammetry (CV) tests were performed. As shown in Fig. 7(a), the CV curves of the CS, ACS-800-1, ACS-800-2, ACS-800-3, and ACS-800-4 electrodes in a 6 M KOH solution demonstrate that KOH doping significantly enhances the electro-chemical performance of the materials. In the electrochemical system, the reduction peak, a key indicator of the reduction capability of the material, is closely related to the formation of H_2_O_2_. After KOH activation, more active sites are generated on the surface of the cathode material, accelerating electron adsorption on the electrode surface and improving the electron transfer efficiency.^50^ The CV curve of ACS-800-3 shows a larger area, indicating its higher electrochemical activity.^51,52^ The utilization of active sites in ACS-800-3 is significantly improved, with enhanced charge transfer efficiency between the cathode and solution, effectively promoting H_2_O_2_ generation and subsequent reactions. Additionally, electrochemical impedance spectroscopy (EIS) further confirms these findings. As shown in Fig. 7(b), the Nyquist plots of the CS, ACS-800-1, ACS-800-2, ACS-800-3, and ACS-800-4 electrodes in 6 M KOH solution indicate that ACS-800-3 has the smallest charge transfer resistance (*R*_ct_) among the samples. This indicates that ACS-800-3 has excellent conductivity, faster reaction rates, and rapid catalytic kinetics, consistent with the CV analysis.

### 3.4 Analysis of electrocatalytic mechanism

Numerous studies indicate that H_2_O_2_ and ˙OH play crucial roles in electrocatalytic processes. In a three-dimensional electrode system, direct and indirect oxidation mechanisms work together to drive the electrochemical degradation of TC, with ˙OH likely dominant in indirect oxidation. H_2_O_2_ is generated *in situ via* O_2_ reduction on the electrode surface,^46,53^ and its O–O bond breaks under catalysis to form oxygen-containing free radicals such as ˙OH, which are key in TC degradation. The reaction rate and final degradation are directly tied to the generation and transformation of these radicals. Insufficient oxygen-containing radicals may halt the reaction, impacting degradation. Thus, ideal cathode materials must have excellent oxygen-radical transformation ability.

To analyse the degradation process, salicylic acid was used as a hydroxyl radical (˙OH) scavenger in the experiment. Hydroxyl radicals, known for their high reactivity, are capable of degrading a variety of organic pollutants through oxidation processes. As observed in Fig. 7(c), the degradation efficiency decreased upon the addition of salicylic acid, indicating the crucial role of ˙OH radicals in the degradation mechanism. The reduction in degradation efficiency suggests that ˙OH radicals are primarily responsible for attacking and breaking down TC molecules. By introducing benzoquinone, a superoxide radical (O_2_˙^−^) scavenger, into the degradation system, it was observed that TC degradation was hardly affected, indicating that O_2_˙^−^ radicals did not play a significant role in this process. This result further emphasizes the importance of ˙OH radicals in the effective degradation of TC, rather than other reactive oxygen species. The experiment also monitored the changes in the content and transformation of H_2_O_2_ and ˙OH.

As shown in Fig. 7(d), the concentration of H_2_O_2_ for ACS-800-3 reached its peak at 40 min. Over time, the H_2_O_2_ concentration gradually decreased, indicating the initial reduction of O_2_ to H_2_O_2_, followed by the reaction of H_2_O_2_ to form ˙OH. The smooth curve suggests that the reaction approached dynamic equilibrium, with the generation and consumption of H_2_O_2_ balanced, and most of it was converted to ˙OH for pollutant removal. Therefore, ACS-800-3 can effectively transform H_2_O_2_ into ˙OH within a certain time, demonstrating good electrocatalytic activity. The yield of ˙OH was indirectly evaluated by observing the changes in the intensity of the characteristic peaks. As shown in Fig. 7(e), ˙OH was indeed generated during the degradation process of ACS-800-3, consistent with the results of the H_2_O_2_ degradation experiment. Furthermore, Fig. 7(f) indicates that the content of ˙OH was assessed by changes in the fluorescence peak intensity. The increasing fluorescence peak intensity over time shows that ACS-800-3 can steadily produce ˙OH to accelerate the decomposition of TC. The highly oxidative ˙OH can effectively oxidize TC adsorbed in the pores of the carbon material, efficiently removing pollutants. The collective evidence from the scavenger experiments and fluorescence spectroscopy strongly indicates that ˙OH is the primary reactive oxygen species responsible for the degradation of TC. Although techniques such as electron spin resonance (ESR) spectroscopy could provide more direct spectroscopic evidence for ˙OH generation, the consistent results from multiple indirect methods employed here offer robust and reliable validation of the proposed mechanism. Future studies will aim to incorporate direct detection techniques such as ESR to further solidify these findings. Fig. 7(g) illustrates the mineralization process of a 25 mg L^−1^ TC solution. The degradation rate of TC remained stable in the first 200 min. Within 360 min, 90% of the total organic carbon (TOC) was removed, proving the excellent TC degradation ability of ACS-800-3. It can transform most of the TC into inorganic substances such as H_2_O, CO_2_, and NH_4_^+^ (eqn (3-1) to (3-6)). Compared to the red curve representing TC degradation, a “saw-tooth” trend was observed, which further confirms the successful mineralization of TC. These results provide new evidence for the efficient treatment of TC.

3-1 O_2_ + * → O_2_*

3-2 O_2_* + e^−^ → O_2_˙^−^

3-3 O_2_˙^−^+ H^+^ → HO_2_*

3-4 HO_2_* + (H^+^ + e^−^) → H_2_O_2_ + *

3-5 H_2_O_2_ + e^−^→˙OH + OH^−^

3-6 ˙OH + TC → CO_2_ + H_2_O + NH_4_^+^ + OH^−^

To investigate the impact of different water bodies on TC degradation, deionized water, Harbin city tap water, and Harbin Songhua River rainwater were selected. As shown in Fig. 7(h), the results indicate that the ACS-800-3 cathodic electrocatalytic material can decompose antibiotics in water bodies and has good electrocatalytic effects, showing potential for the treatment of real wastewater.

### 3.5 Stability study

To evaluate the reusability of ACS-800-3, stability tests were conducted. As shown in Fig. 7(i), the degradation efficiency remained at 89.8% after the first use and stayed as high as 86.7% even after 20 cycles. This indicates the excellent stability and durable degradation efficiency of ACS-800-3. The minimal decline in degradation efficiency, coupled with its remarkable stability, further underscores the promising applicability of ACS-800-3 in electrocatalysis.

## 4. Conclusions

After KOH activation and high-temperature calcination, ACS develops abundant microporous structures and an enlarged specific surface area. As an electrode material for TC electrocatalytic degradation, it shows remarkable redox ability. Experimental data indicate that various parameters, such as calcination temperature, activation ratio, current density, and solution pH, significantly affect the degradation performance of ACS. Thus, optimizing these parameters is crucial for enhancing the TC degradation efficiency of ACS. Notably, ACS-800-3 stands out due to its unique structure, good conductivity, and exceptional redox activity. It effectively promotes H_2_O_2_ and ˙OH generation. The generation of strong oxidative ˙OH radicals is supported by radical quenching tests and fluorescence probing, which can efficiently oxidize TC into intermediates for further decomposition and removal. In experiments on simulated TC wastewater, the ACS-800-3 electrode achieved a TC degradation rate of 90.6% within 200 min. This significant result proves that ACS electrodes are highly efficient and stable for treating TC wastewater, showing great potential in electrocatalytic degradation applications. Based on the findings of this study, future work will focus on: (1) obtaining direct evidence for the radical intermediates using electron spin resonance (ESR) spectroscopy; (2) investigating the degradation pathways of TC through intermediate identification; and (3) scaling up the system for treating real antibiotic wastewater.

## Author contributions

The authors collectively contributed to all aspects of the research, including conceptualization, study design, execution, data acquisition, analysis, and interpretation. Each author participated in drafting, revising, and critically reviewing the manuscript. All authors have approved the final version for publication, consented to submission to this journal, and agree to be accountable for the integrity and accuracy of the work.

## Conflicts of interest

There are no conflicts to declare.

## Data Availability

No additional data are available. All underlying data are available in the article.
